# Phenobarbital‐Induced Toxic Epidermal Necrolysis: A Case Report

**DOI:** 10.1002/ccr3.73048

**Published:** 2026-06-26

**Authors:** Biniyam Tedla Mamo, Solomon Getnet Meshesha, Zelalem Aysheshim Andualem, Kisi Chemeda Gutema, Abenezer Abrham Anito, Mehiret Maru Haile, Hilina Worku Jembere, Asnakech Alemu, Teshita Shute

**Affiliations:** ^1^ Ethiopian Food and Drug Administration Addis Ababa Ethiopia; ^2^ Jimma University, Institute of Health Jimma Ethiopia; ^3^ World Health Organization, Ethiopia Office Addis Ababa Ethiopia

**Keywords:** adverse drug reaction, pharmacovigilance, phenobarbital, severe cutaneous adverse reaction, toxic epidermal necrolysis

## Abstract

Adverse drug reactions (ADRs) can lead to severe consequences and increased morbidity and mortality rates. Phenobarbital is one of the most common anti‐epileptic drugs that has numerous adverse drug reactions, including toxic epidermal necrolysis. The event is rare and a medical emergency. A six‐year‐old female epileptic child with phenobarbital‐induced toxic epidermal necrolysis was referred for the management of diffuse exfoliative lesions involving her eyes and buccal mucosa. Associated with the skin lesions, the patient reported high‐grade fever, reddish eye discoloration, dysphagia and dry cough. The exfoliative skin lesion involved 90% of her total body surface area. The patient was managed with withdrawal of phenobarbital, IV antibiotics, systemic corticosteroid, twice daily wound care, analgesic, and nutritional support. Early recognition, discontinuation of offending medications and prompt intervention are crucial to mitigate harm. Raising awareness about ADRs and their management is vital for enhancing patient safety and outcomes.

## Key Clinical Message

Antiepileptic drugs like phenobarbital can cause toxic epidermal necrolysis (TEN), a rare but life‐threatening medical emergency. Vigilant assessment and continuous monitoring are essential after treatment initiation. Early detection through rigorous patient observation ensures prompt intervention, which is critical for managing these severe adverse reactions and improving clinical outcomes.

**Abbreviations**
ADR: adverse drug reaction
ASM: Anti‐seizure medication
cADRs: Cutaneous adverse drug reactions
ICU: intensive care unit
IV: intravenous
RBC: red blood cell
SJS: Stevens–Johnson syndrome
TEN: toxic epidermal necrolysis
WBC: white blood cell

## 1 Background

Adverse drug reactions (ADRs) are a pivotal contributor to a significant number of morbidity and mortality in the healthcare industry, which manifests as harmful and undesired responses to the drug at prescribed doses. According to the World Health Organization (WHO), adverse drug reactions (ADRs) are defined as Any response to a drug that is noxious and unintended and that occurs at doses typically used in humans for the prophylaxis, diagnosis, or therapy of disease or the modification of physiological function [1]. An anticonvulsant drug that slows brain and nervous system activity, primarily used for seizure control in epilepsy, sedation before surgery and short‐term treatment of sleep disorders. However, phenobarbital can cause serious ADRs such as severe allergic reactions, respiratory depression, severe cutaneous adverse reactions, and hematological disorders [2].

Cutaneous adverse drug reactions (cADRs) associated with Anti‐seizure medication (ASM)therapy are the most prevalent, affecting approximately 8% of the global population. cADRs include maculopapular exanthema (MPE), drug rash with eosinophilia and systemic symptoms (DRESS), Stevens–Johnson syndrome (SJS) and toxic epidermal necrolysis (TEN), the primary reason for discontinuing ASM therapy [2]. Hypersensitivity reactions can occur after beginning treatment with phenobarbital (PB) and other aromatic antiepileptic drugs (AEDs), like carbamazepine (CBZ) and phenytoin (PHT), within a range of 1 to 63 days [3].

Stevens‐Johnson syndrome (SJS) or toxic epidermal necrolysis (TEN) is a spectrum of severe, acute, mucocutaneous, T‐cell mediated delayed type IV hypersensitivity response that is uniformly associated with various medicines and usually manifests 1–3 weeks after the initiation of therapy [4]. The estimated incidence ranges from 1.1 to 6.0 per million. Both SJS and TEN have a low occurrence, but they can cause disability or death, with a 10% to 40% mortality rate [3]. SJS and TEN are rare yet life‐threatening mucocutaneous disorders characterized by epidermal and mucosal necrosis as well as skin detachment. Their differentiation is by the percentage of body area affected, with TEN being the most severe. In SJS, the affected body surface percentage is < 10%; in SJS‐TEN overlap, it is 10%–30% and in TEN, it exceeds 30% [3]. The risk factors for TEN/SJS include having a history of allergies, old age, having an HIV infection, pre‐existing liver disease and having chronic underlying conditions [3]. Recent studies have shown that different ethnic populations may have dissimilar risks regarding developing anti‐epileptic drug (AED) induced adverse reactions due to various genetic backgrounds [5]. The aim of this study is to assess the clinical presentation and management of a life‐threatening case of phenobarbital‐induced toxic epidermal necrolysis. It specifically highlights the importance of early recognition, multidisciplinary intervention and vigilant ADR assessment to improve patient safety and outcomes following unintended severe adverse drug reactions.

## 2 Case Presentation

**FIGURE 1:**
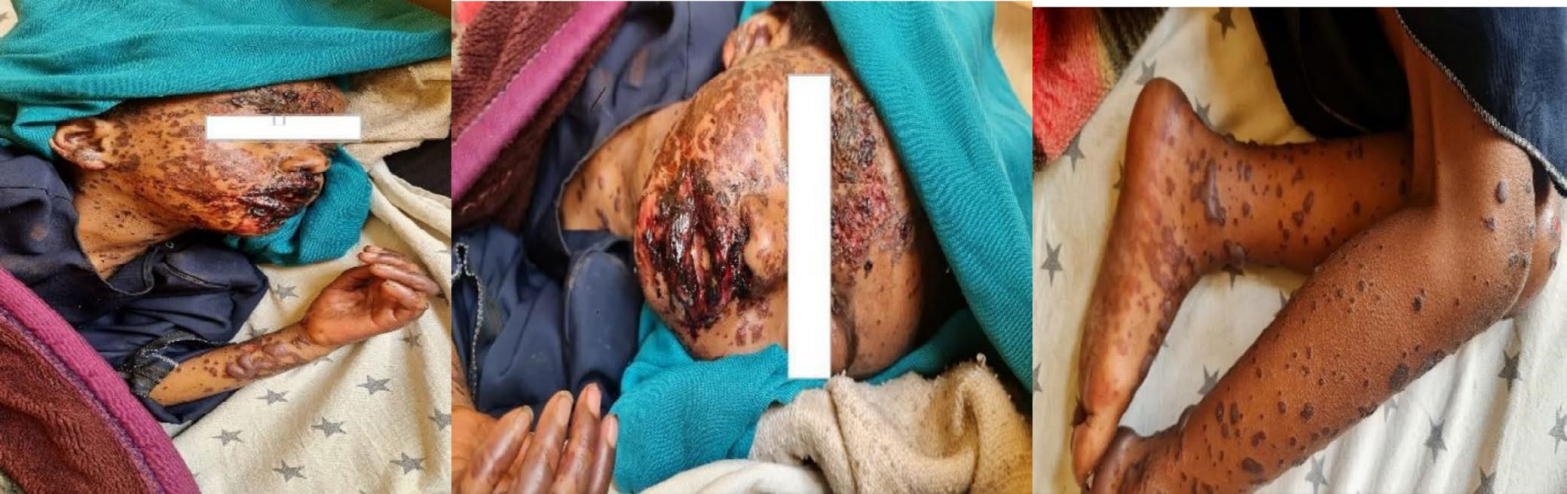
(a–c) Showing ruptured blisters and extensive skin exfoliation involving the face, oral cavity, and extremities.

This is a six‐year‐old female patient; she has been known to have epilepsy for the past one year. She took phenytoin 20 mg PO twice a day for the past 9 months. Her medication was changed to phenobarbital 50 mg PO BID at her follow‐up clinic, as the previous medication was not available at the hospital. Eighteen days after the initiation of phenobarbital, the patient developed a diffuse skin rash with blistering skin eruption involving most of her body (face, chest, abdomen, back, and legs). Subsequently, the lesion became exfoliated unevenly. Associated with her skin symptoms, she had a history of fever, reddish discoloration of the eye, dry cough and difficulty swallowing. Upon presentation, her pulse rate was 126 beats per minute, respiratory rate was 28 breaths per minute, temperature was 38.6°C, and oxygen saturation was 96% on atmospheric air. On examination, there was also crusted plaque with extensive lip ulcer involving the oral mucosa and tongue. The lesion was oozing blood. Diffused purpuric and exfoliative lesions involved 90% of the total body surface area, including her palms and soles. Along with this, there were blistering skin lesions distributed on her abdomen, thighs, and legs. This patient had no history of allergy and was not taking medication other than the phenobarbital. See Figure 1a–c.

### 2.1 Laboratory Investigations and Treatment

Laboratory results were unremarkable except slight elevation of ESR and prolongation of the coagulation profile (Table 1). In the present case, the skin biopsy was not done. The patient was admitted to the pediatric ward with a diagnostic consideration of phenobarbital‐induced toxic epidermal necrolysis. The phenobarbital was discontinued, and she was managed with IV fluid, IV antibiotics (Ceftazidime and vancomycin) and a topical antibiotic (Fusidic acid 2% cream) used on the necrotic area of the skin. IV analgesic (morphine), IV corticosteroid (hydrocortisone), twice‐daily wound care and nutritional support were provided. The causality assessment by the Ethiopian national pharmacovigilance advisor committee for the drug was done based on WHO‐UMC categories. The causal association between the event and the drug was classified as certain.

**TABLE 1:** Summary of laboratory results.

Laboratory result	At admission	After 2 days	After 2 weeks	Reference
*Blood tests*				
Hgb (g/dL)	12.1	10.8	12	11–17.5
WBC	4.9	6.1	6	3.0–10.0
Platelet count	548	456	278	150–450
MCV (fL)	78.7	78.7	78	79–95
RBS (mg/dL)	121	—	—	< 200
Sodium (mmol/L)	140	135	140	136–145
Potassium (mmol/L)	3.5	3.54	4.9	3.5–5.1
I Calcium (mmol/L)	1.94	—	—	2.1–2.5
Chloride (mmol/L)	103	102.8	109	98–107
ALT (mg/dL)	20	—	—	0–35
AST (mg/dL)	22	—	—	0–41
Creatinine (mg/dL)	0.17	—	—	0.7–1.2
BUN (mg/dL)	13	—	—	06–22.0
PT	17.6	—	—	10–14 s
aPTT	40	—	—	21–35 s
INR	1.5	—	—	—
ESR	20	—	—	0–10 mm/h
*Urinalysis*				
Urine WBC	0–5 WBC/hpf	—	—	0–5 WBC/hpf
Urine RBC	0–2 RBC/hpf	—	—	0–3 RBC/hpf
Urine glucose	Negative	—	—	—
Urine ketone	Negative	—	—	—
Urine protein	Negative	—	—	—

Abbreviations: ALT, Alanine Transaminase; aPTT, Activated Partial Thromboplastin Time; AST, aspartate aminotransferase; BUN, blood urea nitrogen; Hgb, hemoglobin; INR, International Normalized Ratio; MCV, mean corpuscular volume; PT, prothrombin time; RBC, red blood cell; WBC, white blood cell.

### 2.2 Outcome and Follow‐Up

**FIGURE 2:**
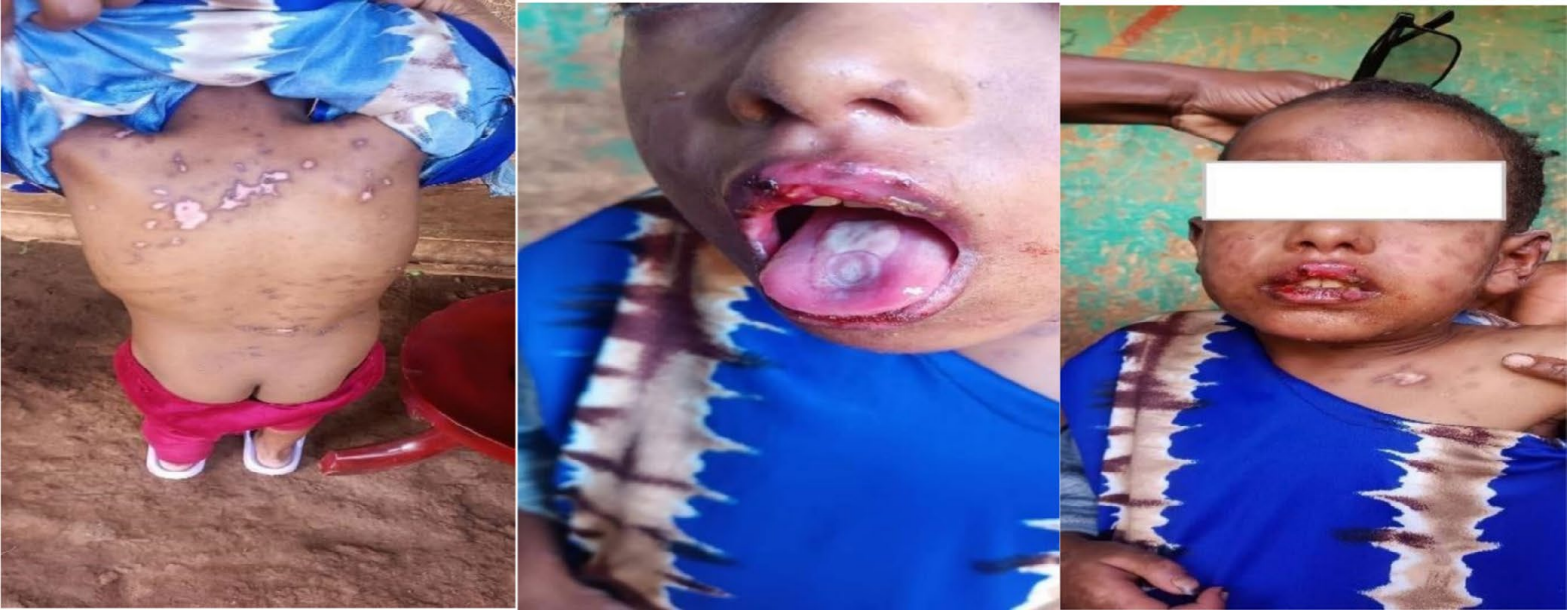
(a, b, c) Figure showing healing of exfoliating skin lesion on anterior chest, upper and middle back, face and oral mucosa that involved lip and tongue.

The clinical prognosis of the case was good with a predicted mortality rate of 12% based on the SCORETEN clinical assessment (Table 2). In this case, a heart rate greater than 120 beats per minute and a large body surface area (BSA) greater than 10% were predictive factors of mortality. The patient's condition progressively improved, the skin lesion also started to heal and her vital signs were progressively normalized. After 21 days of hospital admission and treatment, the patient was discharged. On subsequent follow‐up (after 1 month), the lesions on her face, chest, abdomen, back, and legs were healed; there was a partially healed oral mucosa lesion involving her tongue (Figure 2a–c). Upon follow‐up, her complaint was pain during swallowing and eye pain. Her parents were advised to give liquid and semi‐liquid foods; additionally, oral care was also recommended. Furthermore, she was linked to the ophthalmologic unit for additional eye evaluation.

**TABLE 2:** SCORETEN mortality rate prediction of the patient.

Prognostic factors	Score	Mortality rate
Age > 40 years	0	Score 0 = 1%–3% Score 2 = 12% Score 3 = 35% Score 4 = 58% Score 5 or more = 90%
Heart rate > 120 beats per minute	1	
Cancer or hematologic malignancy	0	
Involved body surface area > 10%	1	
Blood urea nitrogen level > 10 mmol/L (28 mg/dL)	0	
Serum bicarbonate level < 20 mmol/L (20 mEq/L)	0	
Blood glucose level > 14 mmol/L (252 mg/dL)	0	

## 3 Discussion

Stevens‐Johnson Syndrome (SJS) and Toxic Epidermal Necrolysis (TEN) are severe Type IV hypersensitivity reactions characterized by T‐cell‐mediated delayed hypersensitivity. This immunologic response is typically triggered by pharmacological agents or less frequently by infections; however, some cases remain idiopathic in origin. Pathophysiologically, SJS/TEN is defined by extensive, immune‐mediated apoptosis of keratinocytes, resulting in widespread necrosis of the skin and mucosal membranes.

The study highlighted a child who presented with an extensive exfoliative skin rash following phenobarbital, likely toxic epidermal necrolysis (TEN) and predictors of mortality. This result was in line with previous studies that report that anti‐epileptic drugs (AED) were significantly associated with severe skin reactions, including toxic epidermal necrolysis [6, 7]. It is therefore crucial to take a careful approach when prescribing medications linked to TEN/SJS for patients with seizure disorder.

The patient presented with extensive exfoliative lesions with diffused blistering and bullae along with crusted and necrotic plaques, involving the oral mucosa and lips. This clinical presentation is similar to cases presented with phenobarbital‐related severe cutaneous adverse reactions [8, 9]. The estimated incidence of toxic epidermal necrolysis ranged between 0.4 cases per million [10]. The increasing incidence of SJS/TEN with age is likely due to more frequent drug prescriptions and comorbidities that alter the drug metabolism and final medication effects [10]. Children have lower mortality rates than adults; however, it is observed that the incidence of long‐term complications in the pediatric population is high [11].

According to clinical scoring, the mortality rate of this case was 12%. The various studies showed that the mortality of toxic epidermal necrolysis ranged from 10% to 40%, even though the mortality in adult and elderly patients was very high compared with pediatric age groups [4].

Identifying the drugs that cause SJS/TEN is a key factor in preventing these conditions. While the list of medications that can induce SJS/TEN is extensive and in theory, any drug can lead to these conditions, only a small number of drugs account for most cases, particularly among children. Thus, the majority of cases of drug‐induced SJS/TEN are linked to certain classes of medications, such as anticonvulsants, antibiotics, nonsteroidal anti‐inflammatory drugs (NSAIDs) and allopurinol [12, 13]. Hence, prior to the prescription of these drugs that can cause SJS/TEN, it is crucial to inquire about the patient's history of drug allergies, atopic conditions and familial allergy history. This will help prevent the onset of delayed hypersensitivity reactions like TEN.

The primary management of toxic epidermal necrolysis or severe cutaneous adverse reactions is to discontinue the suspected drugs. Transferring the patient to the burn or ICU and the treatment requires a multidisciplinary approach. The management depends on the extent of skin lesions and body surface area. Generally requires adequate IV fluid treatment, pain control, IV antibiotics, anti‐acids for gastric prophylaxis and intensive skin care management [14]. The treatment of TEN with steroids is controversial. The studies showed that steroids increased the risk of mortality, sepsis, prolonged hospital stay, and poor wound healing. A recent case–control study done in Europe showed that steroids did not show a significant effect on mortality in comparison with supportive care only [15]. Beyond the individual case management, the study uses the data to advocate for broader pharmacovigilance, highlighting the need for increased awareness regarding common drugs like phenobarbital and their potential for rare, catastrophic adverse reactions. A study has a limitation in assessing the skin biopsy to confirm the suspected clinical cases because of the unavailability of this kind of diagnostic test in the hospital.

## Author Contributions

**Biniyam Tedla Mamo:** conceptualization, data curation, methodology and writing original draft. **Solomon Getnet Meshesha:** conceptualization, data curation, methodology. **Zelalem Aysheshim Andualem:** writing – review and editing. **Kisi Chemeda Gutema:** writing – review and editing. **Abenezer Abrham Anito:** writing – review and editing. **Mehiret Maru Haile:** writing – review and editing. **Hilina Worku Jembere:** writing – review and editing. **Asnakech Alemu:** writing – review and editing. **Teshita Shute:** writing – review and editing.

## Funding

The authors have nothing to report.

## Ethics Statement

The authors have nothing to report.

## Consent

Written Informed consent was obtained from the patient's family for publication of this case report and any accompanying images.

## Conflicts of Interest

The authors declare no conflicts of interest.

## Data Availability

The authors have nothing to report.
